# The Brain Microvascular Endothelium Supports T Cell Proliferation and Has Potential for Alloantigen Presentation

**DOI:** 10.1371/journal.pone.0052586

**Published:** 2013-01-08

**Authors:** Julie Wheway, Stephanie Obeid, Pierre-Olivier Couraud, Valery Combes, Georges E. R. Grau

**Affiliations:** 1 Discipline of Pathology, Sydney Medical School, University of Sydney, Camperdown, New South Wales, Australia; 2 Unité 1016, Institut National de la Santé et de la Recherche Médicale, Institut Cochin, Paris, France; 3 Unité Mixte de Recherche 8104, Centre National de la Recherche Scientifique, Paris, France; 4 Université Paris Descartes, Paris, France; INSERM, France

## Abstract

Endothelial cells (EC) form the inner lining of blood vessels and are positioned between circulating lymphocytes and tissues. Hypotheses have formed that EC may act as antigen presenting cells based on the intimate interactions with T cells, which are seen in diseases like multiple sclerosis, cerebral malaria (CM) and viral neuropathologies. Here, we investigated how human brain microvascular EC (HBEC) interact with and support the proliferation of T cells. We found HBEC to express MHC II, CD40 and ICOSL, key molecules for antigen presentation and co-stimulation and to take up fluorescently labeled antigens via macropinocytosis. In co-cultures, we showed that HBEC support and promote the proliferation of CD4^+^ and CD8^+^ T cells, which both are key in CM pathogenesis, particularly following T cell receptor activation and co-stimulation. Our findings provide novel evidence that HBEC can trigger T cell activation, thereby providing a novel mechanism for neuroimmunological complications of infectious diseases.

## Introduction

The induction of adaptive cellular immunity is a function of professional antigen presenting cells (APCs) such as dendritic cells, which provide signal 1 (peptide-major histocompatibility complex (MHC)), signal 2 (co-stimulatory molecules), and signal 3 (instructive cytokines) to naive T lymphocytes upon antigen encounter [1].

Endothelial cells (EC) form the inner lining of blood vessels and are positioned between circulating lymphocytes and peripheral tissues. As such, EC are the first cells with which T cells come into direct contact in the circulation. The hypothesis that EC may be able to act as APC is based upon the intimate interactions between EC in microvessels and T cells during transendothelial migration to lymph nodes or peripheral tissues. That is, EC may acquire antigenic proteins and present them on MHC class I and II molecules at their apical surface. The vascular EC that separate the blood stream from the brain parenchyma is referred to as the blood brain barrier (BBB). The BBB provides both anatomical and physiological protection for the central nervous system, regulating the entry of many substances and blood borne cells into the nervous tissue. There is increasing evidence of interactions between T cells and brain endothelium in diseases such as multiple sclerosis, cerebral malaria (CM) and viral neuropathologies. Of particular note, the diameter of microvessels, where the pathology is seen during CM, is smaller than the size of activated lymphocytes; therefore the latter physically “brush” the EC surface and can thus interact very closely. Additionally, during CM, both T cells and monocytes are arrested in brain microvessels [2] and we recently demonstrated that brain EC can display antigens from infected erythrocytes on their surface, thereby possibly initiating immune responses [3].

MHC expression, which is the primary requirement for APC activity has been demonstrated on EC with both MHC I and II upregulated following cytokine treatment [4]–[6]. Moreover, EC may also qualify as APCs due to the secretion of cytokines, particularly GM-CSF [7], [8]. Some studies using MHC matched donors supports the model that cultured human EC are able to present antigen and thus re-activate primed CD4^+^ T cells [9]–[11]. However, EC are specifically able to re-stimulate T cells, but not to prime naïve T cells, which is a hallmark of “professional” APCs such as dendritic cells [12]–[14]. Additional studies using co-cultures of MHC-mismatched EC and T cells resulted in the activation of both CD4^+^ and CD8^+^ T cells suggesting that EC are able to present alloantigens [15], [16].

The body of evidence supporting the role of EC as APC (reviewed in [17]) led us to investigate the capacity of brain microvascular EC to act as APC and modulate T cell activation and proliferation. Here we confirm and expand on previous data [18] and show that immortalised human brain microvascular hCMEC/D3 endothelial cells (HBEC) express MHC II and the co-stimulatory molecules CD40 and ICOSL following cytokine stimulation. We also demonstrate that HBEC were able to take up fluorescently labeled antigens via macropinocytosis and clathrin coated pits. Moreover in our peripheral blood mononuclear cell (PBMC)/HBEC co-cultures, HBEC support and promote the proliferation of both CD4^+^ and CD8^+^ T cells suggesting that the brain endothelium is able to process and present antigens to allogeneic T cells. Finally, we were able to demonstrate that the interaction between T cells and HBEC occurs in a 2-way fashion as the expression of MHC II on HBEC was significantly increased following co-culture with PBMC. Combined, our data indicates that EC can act as semi-professional APC, which has important implications for the presentation of antigens to T cells, resulting in the activation of the effector T cell response in neuroinfectious diseases, particularly CM.

## Materials and Methods

### Ethics Statement

The blood samples used in this study are from anonymous donors from the Australian Red Cross Blood Bank. Protocol was approved by the University of Sydney Human Ethics Committee (Approval #10218).

### Cells and cell culture

Immortalised human brain microvascular hCMEC/D3 endothelial cells (HBEC) [18] were cultured in EBM-2 medium (Lonza CC-3156). Cells were grown on plates pre-coated with rat tail collagen type I (BD Biosciences). Cytokine activation of HBEC was performed by treating the cells with 10 ng/ml TNF or 50 ng/ml IFNγ (Peprotech) for 18 h.

### Human PBMC preparation

PBMC were separated either from leukopacks or from heparinized venous blood by conventional Ficoll gradient and brought to 2×10^6^/ml in complete medium. PBMC were frozen in 10% DMSO in FCS and stored in liquid nitrogen. PBMC were thawed and washed twice in cold medium before use in assays.

### T cell isolation and CFSE staining

CD4^+^ and CD8^+^ T cells were isolated from freshly thawed PBMCs using an Easysep® (Stemcell Technologies) negative selection kit according to the manufacturer's instructions. For labeling both isolated T cells and whole PBMCs with Carboxyfluorescein succinimidyl ester (CFSE; Invitrogen), cells (at a density of 10^7^ cells/ml) were incubated for 10 min at 37°C in 5 mM CFSE in serum free RPMI. The labelling reaction was stopped by the addition of serum. Cells were then washed 3 times prior to use. For the quantification of cell proliferation, cells were analysed by flow cytometry with a reduction in CFSE MFI indicative of cell division.

### Flow cytometry

For multicolor flow cytometric analysis, HBEC were incubated in the presence of fluorochrome-conjugated mAbs against CD105 (SN6), CD106 (STA), CD80 (2D10.4), CD86 (IT2.2), CD40 (5C3), HLA-DR/MHC II (LN3) and CD275 (MIH12) (all from eBioscience), CD54 (5.6E; Beckman Coulter) and β_2_-microglobulin/MHC I (TÜ99; BD Biosciences) as per manufacturer's instructions.

### Antigen uptake analysis

The ability of HBEC to take up fluorescently labeled protein was assessed using flow cytometry after the cells were incubated with either 1 mg/ml Fluorescein isothiocyanate (FITC)-Ovalbumin (OVA) or Lucifer Yellow (Invitrogen) at 37°C for 45 min and washed three times with PBS. Results are expressed as the percentage increase in mean fluorescence intensity (MFI), which subtracts any fluorescence detected by nonspecific surface binding after incubation on ice. The percentage increase in MFI is calculated as follows; % increase in MFI = [(uptake at 37°C)/(uptake at 4°C)×100]. To selectively inhibit macropinocytosis and other actin-dependent mechanisms, HBEC were pre-incubated for 15 min at 37°C with cytochalasin D (CCD; 10 µM; Sigma).

### Conjugation assays

The ability of HBEC to form long-lasting conjugates with T cells was assessed using an *in vitro* conjugation assay. Briefly, CD4^+^ and CD8^+^ T cells were isolated from PBMC using EasySep®. Isolated T cells and trypsinated HBEC were then labeled with the membrane-labeling agents, PKH26 (red) and PKH67 (green) respectively (Sigma). 1×10^5^ T cells and 1×10^5^ HBEC were co-incubated for 30 min at 37°C prior to flow cytometric analysis. Conjugates were deemed to be positive for both PKH26 and PKH27.

### In vitro T cell proliferation assays

HBEC were cultured to confluence in 24 well tissue culture plates (Corning). Cells were either left under resting conditions or stimulated with a combination of 10 ng/ml TNF and 50 ng/ml IFNγ for 18 h. 1×10^5^ CFSE-labeled PBMCs were added per well with the following conditions; PBMC alone, 0.3 mg/ml αCD3 (eBioscience; Clone HIT3a) or 0.3 mg/ml αCD3+1 mg/ml αCD28 (eBioscience; Clone CD28.2). The co-cultures were incubated for 6 days at 37°C. After 6 days in culture the non-adherent cells were then collected for staining and flow cytometric analysis. Non-adherent cells were stained with PE conjugated anti-human CD4 (eBioscience; Clone OKT4) and PE-Cy5 anti-human CD8a (Biolegend; Clone HIT8a) prior to multi-colour flow cytometric analysis. T cell proliferation was then quantitated with the parameters set to a log scale. A forward scatter vs FL1 was used to gate on the PBMC population that was positive for CFSE. This gated population was then used to differentiate between CD4^+^ T cells and CD8^+^ T cells. CFSE histograms depict the number of events (y-axis) and the fluorescence intensity (x-axis) with proliferating cells displaying a progressive 2-fold loss in fluorescence intensity following cell division, indicative of proliferating cells. To determine whether cell contact is necessary for EC to support T cell proliferation, the use of transwells was employed. 1×10^5^ PBMC/well were placed in 0.4 µm transwells (Costar) and co-culture with HBEC performed as outlined above.

### MHC II expression on HBEC following co-culture

To assess the expression of MHC II on HBEC following co-culture with PBMC, HBEC were removed by trypsinisation following 6 d of co-culture. HBEC were then stained with anti-human MHC II (HLA-DR; eBioscience). For flow cytometric analysis CFSE positive cells (PBMC) were excluded by gating to ensure MHC II analysis was conducted on HBEC only.

## Results

### HBEC express key molecules for antigen presentation and T cell activation

For this study we employed a particular line of immortalized human microvascular EC (HBEC; hCMEC/D3) that recapitulates many of the key characteristics of primary brain EC and thus has been validated as an excellent model of the BBB for *in vitro* studies [18]–[20].

**Figure 1:**
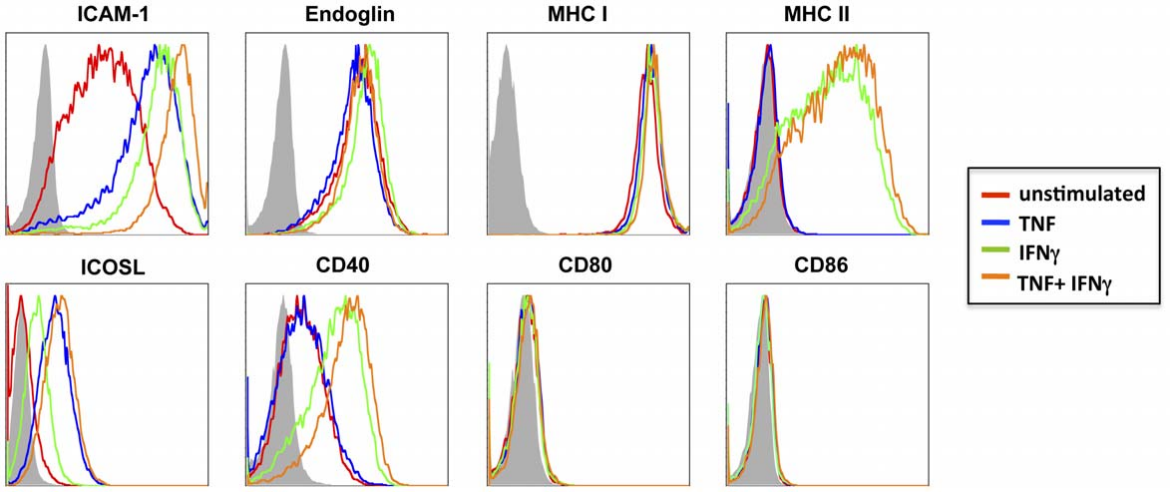
Expression of markers relevant to antigen presentation and T cell activation on HBEC. Histograms represent flow cytometry results from unstimulated and cytokine stimulated HBEC cells 18 h following stimulation. HBEC were stimulated with either 10 ng/ml TNF (blue line), 50 ng/ml IFNg (green line), or 10 ng/ml TNF+50 ng/ml IFNg (orange line) and compared to unstimulated cells (red line). Cells were stained with mAbs against CD54 (ICAM-1), Endoglin (CD105), MHC II (HLA-DR), ICOSL (CD275), CD40, CD80 and CD86 as per manufacturers instructions. Data are representative of four independent experiments.

A number of adhesion molecules known to be expressed by brain endothelium are involved, under inflammatory conditions, in the migration of activated leukocytes across the BBB. Flow cytometric analysis of HBEC cells not only confirmed the strong basal expression of ICAM-1, but also demonstrated a marked up-regulation following stimulation with TNF and/or IFNγ (Fig. 1). Endoglin (CD105), an EC marker predominantly expressed by proliferating EC was expressed at high levels basally, with no regulation in expression seen following pro-inflammatory cytokine stimulation (Fig. 1). Similarly, MHC I (β_2_-microglobulin) was expressed at high levels basally on HBEC with no increase observed following cytokine stimulation (Fig. 1). This is in contrast to previous results whereby MHC I expression has been shown to be upregulated by stimulation with IFNα, -β or –γ [21]. Despite this, our results provide evidence that HBEC, like most cell types, possess the minimal requirement for antigen presentation to CD8^+^ T cells. In contrast to MHC I, despite the low basal expression of MHC II on HBEC cells, its expression was greatly increased upon the addition of IFNγ or TNF+IFNγ (Fig. 1), highlighting a potential role for these cells in antigen presentation to CD4^+^ T cells. Previous analysis of MHC II on EC has proved difficult *in vivo*, with constitutive expression only detected in post-capillary venules [22]. Whilst the expression of the co-stimulatory molecules CD80/CD86 (B7-1/B7-2) was not detected on resting or cytokine-stimulated HBEC cells, the co-stimulatory molecule, CD40 was detected following stimulation with IFNγ or TNF+IFNγ (Fig. 1), indicating that like MHC II the expression is regulated by IFNγ. Previously, CD40 has been demonstrated to be constitutively expressed on primary human brain ECs, with this expression being upregulated following cytokine stimulation [23]. The expression of this co-stimulatory molecule on brain EC provides key evidence for their potential role as APC as the binding of CD40L on helper T cells to CD40 activates ‘APCs’ to upregulate the expression of more co-stimulatory molecules, increase cytokine expression and promote T cell differentiation [24]. Finally, inducible co-stimulator ligand (ICOSL) expression was detected on HBEC following TNF stimulation (Fig. 1). ICOS and its ligand, ICOSL are members of the CD28 family of co-stimulators mediating effector T cell differentiation [25]. Previously, ICOSL has been detected not only basally on human umbilical vein ECs but also upregulated by cytokine stimulation [25], [26].

### HBEC take up antigens using macropinocytosis and clathrin-coated pits

**Figure 2:**
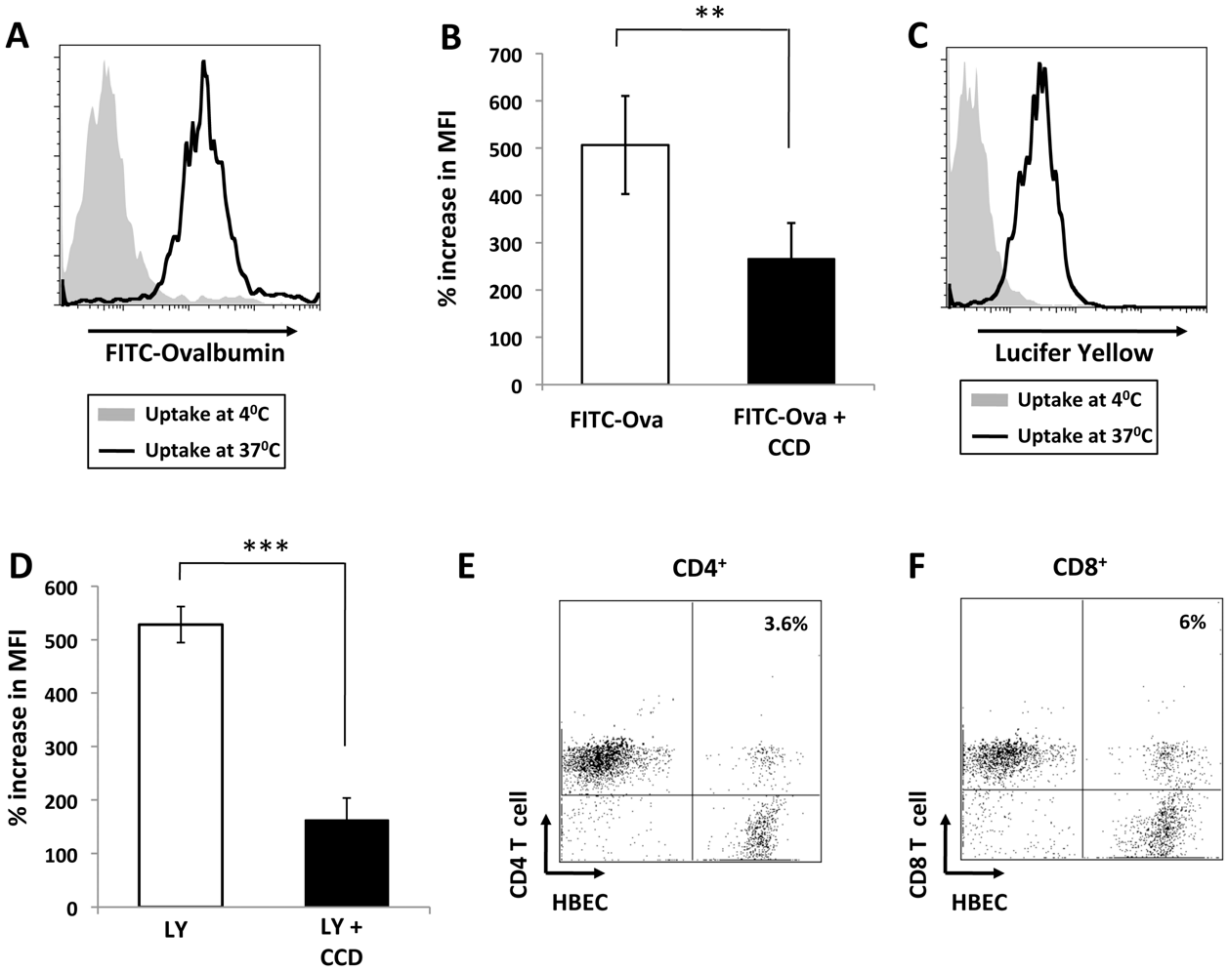
HBEC take up fluorescently labelled antigen via actin-dependent mechanisms and form conjugates with T cells. Flow cytometry histograms depicting level of uptake of FITC-OVA (*A*) and Lucifer yellow (*C*) by HBEC at 37°C (blue line) vs background uptake at 4°C (red line). Data are representative of three independent experiments. Inhibition of FITC-OVA (*B*) and Lucifer yellow (*D*) uptake by HBEC cells pre-incubated with 10 mM Cytochalasin D (CCD). *C*, Flow cytometry histogram depicting level of uptake of Lucifer yellow by HBEC at 37°C (blue line) vs background uptake at 4°C (red line). Data are representative of three independent experiments Percentage increase in mean fluorescence intensity (MFI) is calculated as follows: (MFI following uptake at 37°C/MFI following uptake at 4°C)×100. Data are pooled from three independent experiments (n = 3 per experiment) and are expressed as mean +/− SD. ** and *** indicates statistically significant differences between control and CCD treatment as assessed by Student t test (p, 0.001, p<0.001 respectively). Representative flow cytometry plots indicating the levels of conjugation between HBEC and CD4^+^ *(E)* and CD8^+^ *(F)* cells. HBEC were labeled with PKH67 and isolated T cells labeled with PKH26 and equal numbers of cells were co-cultured for 30 min prior to flow cytometric analysis.

A recent study from our laboratory demonstrated that during malaria, the transfer of parasite antigens to ECs can take place [3], however, the precise mechanisms behind this remain unclear. The ability of our HBEC to take up soluble antigens was assessed *in vitro* using fluorescently labeled antigens in a classic antigen uptake experiment. The ability of HBEC to take up antigen via macropinocytosis and clathrin-coated pits was assessed using Lucifer yellow and FITC-OVA respectively. The amount of fluorescence incorporated into the cells at 37°C was measured by flow cytometry with nonspecific surface binding subtracted following incubation on ice. Interestingly, HBEC were able to take up FITC-OVA via clathrin-coated pits and macropinocytose Lucifer yellow (Fig. 2A, C respectively). To further prove that the uptake of antigen by HBEC was not an experimental artifact, a specific inhibitor of macropinocytosis and other actin-dependent mechanisms, cytochalasin D (CCD; 10 mM) was employed [27]. Indeed, following pre-incubation with CCD, both the uptake of FITC-OVA and Lucifer yellow was significantly inhibited (Fig. 2 B, D) indicating that HBEC have the capacity to take up soluble antigen in a similar manner as professional APC.

### HBEC support the proliferation of activated T cells

As optimal T-cell activation and differentiation *in vivo* requires long-lasting T–APC interaction, a classical *in vitro* conjugate forming assay was adapted to assess the ability of HBEC to form conjugates with T cells [28]. Red fluorescently labeled (PKH26) CD4^+^ or CD8^+^ T cells were incubated in suspension with green fluorescently labeled (PKH67) HBECs with the adherence between HBEC and T cells examined using flow cytometry. Conjugates were determined to be cells positive for both PKH26 and PKH67. Interestingly, both CD4^+^ and CD8^+^ T cells form conjugates, i.e. cell doublets in suspension, with control (data not shown) and cytokine activated HBECs, as shown by flow cytometry (Fig. 2E, F respectively). There were higher percentages of T cell/HBEC conjugates seen when the HBEC were cytokine activated (3.6 vs 1.4% for CD4^+^ and 6.3 vs 2.1% for CD8^+^).

**Figure 3:**
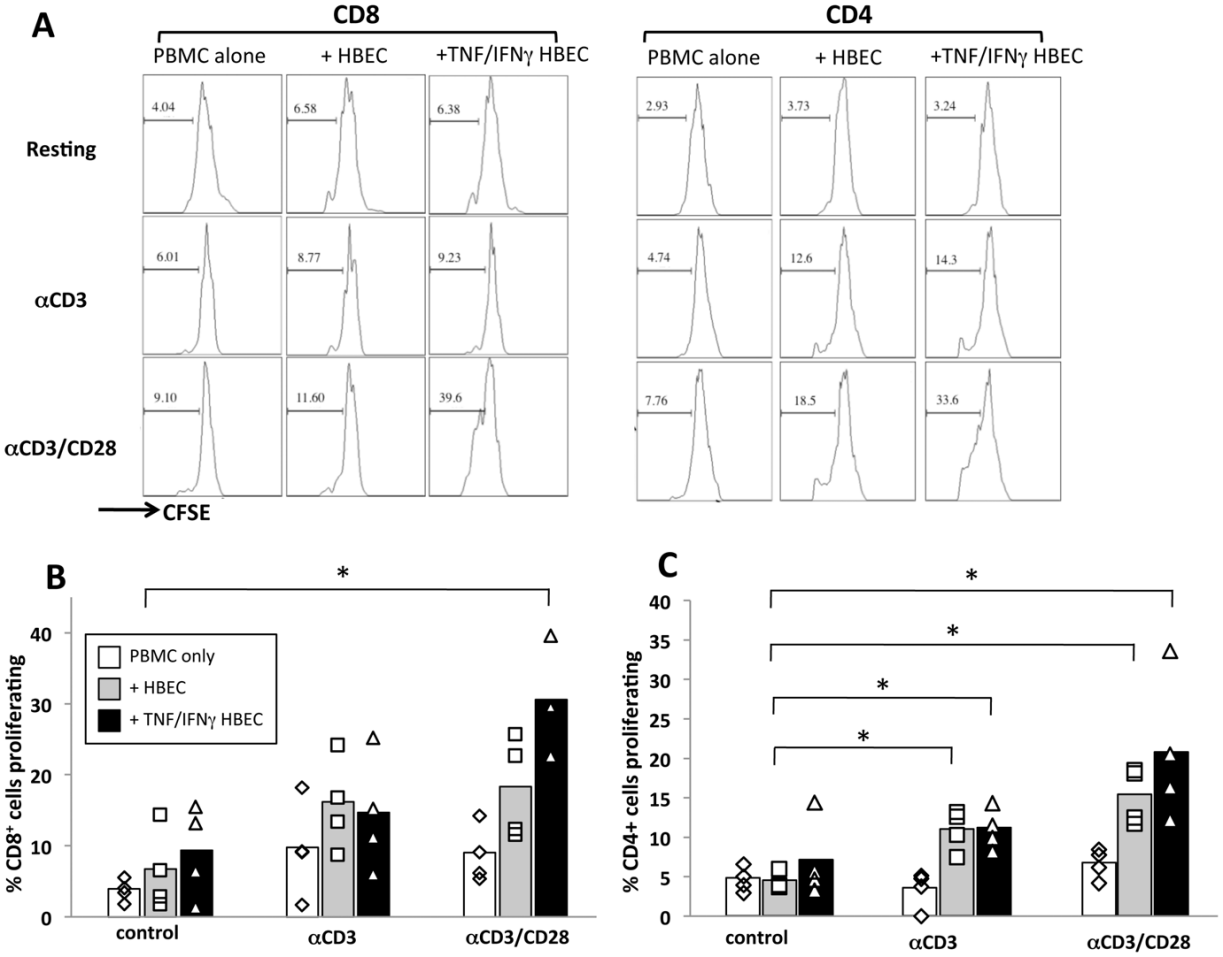
HBEC support the proliferation of CD4^+^ and CD8^+^ T cells. *A*, CFSE histogram plots of gated CD4^+^ (left panel) and CD8^+^ (right panel) 6 days following the start of the co-culture of HBEC and donor PBMC. For co-culture 1×10^5^ CFSE-labelled donor PBMC were co-cultured or not with a confluent monolayer of either resting or 10 ng/ml TNF+50 ng/ml IFNγ pre-stimulated HBEC cells. PBMC were either subjected to resting conditions or stimulation with *a*CD3 or *a*CD3/CD28 mAbs. Following 6 days of culture, cells were harvested and stained with CD4 and CD8 mAbs to identify proliferating cell populations. CFSE histograms depict the number of events (y-axis) and the fluorescence intensity (x-axis) with proliferating cells displaying a progressive 2-fold loss in fluorescence intensity following cell division, indicative of proliferating cells. Histograms are representative of four independent experiments with the same donor. Graphical representation of the percentage of CD4^+^ (*B*) and CD8^+^ (*C*) PBMC proliferating following 6 days of culture either alone (white bars) or in the presence of resting (grey bars) or cytokine stimulated (black bars) HBEC as outlined above. Data is pooled from four independent experiments with the same donor. * indicates statistically significant differences between control PBMC and respective co-culture conditions using a non-parametric Mann-Whitney test (p<0.05).

After determining that HBEC were capable of binding to both CD4^+^ and CD8^+^ T cells, the ability of HBEC to support T cell proliferation and present alloantigens was assessed by co-culturing CFSE-labelled donor PBMCs with a confluent monolayer of either resting or cytokine stimulated HBECs. In addition, the agonistic antibodies αCD3/αCD28 were also added to the assay to mimic T cell receptor (TCR) stimulation and co-stimulation respectively [29]. Six days following co-culture the percentage of CD4^+^ and CD8^+^ T cells proliferating was determined by measuring the reduction in CFSE MFI (Fig. 3A). While the presence of soluble αCD3 and αCD28 resulted in a modest increase in proliferating CD8^+^ cells, the only significant increase in proliferation was observed when the PBMC were co-cultured with TNF+IFNγactivated HBEC and αCD3/αCD28 (Fig. 3B), indicating that HBEC support the proliferation of CD8^+^ T cells, however, the CD8^+^ cells must also be activated via their TCR. Interestingly, CD4^+^ T cell proliferation was significantly increased in the presence of both resting and cytokine-stimulated HBEC (Fig. 3C), however, the CD4^+^ cells also must be stimulated via their TCR with αCD3 or αCD3/αCD28 to observe the HBEC-mediated support of proliferation. It is most likely that the modest increase in proliferation for both CD4^+^ and CD8^+^ T cells following αCD3 stimulation is indicating that the cells were not stimulated using a solid phase activation, i.e. plate bound αCD3.

Experiments using transwells have indicated that when the PBMC were physically separated from the HBEC monolayer during co-culture, the increase in proliferation over control samples were greatly reduced (Fig. S1). This was observed for both CD4^+^ and CD8^+^ T cells suggesting that direct interaction between HBEC and T cells is required for HBEC-mediated support of T cell proliferation.

### MHC expression on HBEC is upregulated following co-culture with allogeneic PBMC

**Figure 4:**
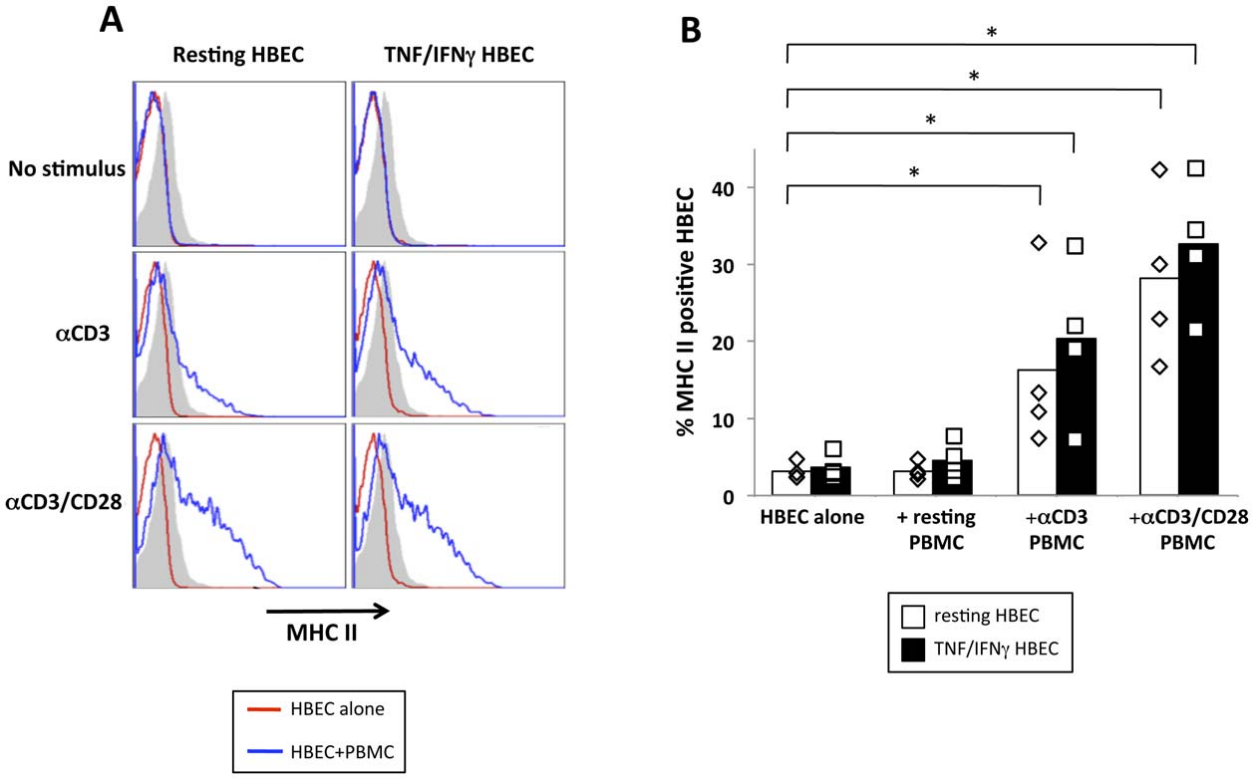
PBMC modulate MHC II expression on HBEC following co-culture. *A*, Histogram plots of HBEC depicting expression of MHC II (HLA-DR) 6 days following the start of the co-culture with donor PBMC. 1×10^5^ CFSE-labelled donor PBMC were co-cultured with a confluent monolayer of either resting (left panels) or 10 ng/ml TNF+50 ng/ml IFNγ pre-stimulated (right panels) HBEC cells. PBMC were either subjected to resting conditions or stimulation with *a*CD3 or *a*CD3/CD28 mAbs (top, middle lower panels respectively). Histograms are representative of four independent experiments with the same donor. *B*, Percentage of MHC II+ HBEC in resting (white bars) vs TNF/IFNγ stimulated (black bars) HBEC. Data is pooled from four independent experiments with the same donor. * indicates statistically significant differences between control HBEC and respective co-culture conditions using a non-parametric Mann-Whitney test (p<0.05).

To determine whether the interaction between T cells and HBEC occurs in a two-way fashion, the expression of MHC II on the HBEC monolayer was determined following 6 days of co-culture with PBMCs. A significant increase in MHC II-positive cells was observed when HBEC were co-cultured with αCD3 orαCD3/αCD28 stimulated PBMCs when compared to HBEC cells alone (Fig. 4A, B) indicating that the donor PBMC were able to modulate the MHC II expression on the HBEC themselves. These conjugates likely involve interactions of ICAM-1/LFA-1 and VCAM-1/VLA-4 on EC/T cells respectively in addition to interactions required for antigen presentation.

## Discussion

In this study, we provide for the evidence that microvascular brain EC are able to act as APCs. Our analysis of MHC and co-stimulatory molecule expression on HBEC show for the first time that brain EC are endowed a “professional” costimulatory ligand of the B7 family, ICOSL. This in conjugation with the expression of MHC II and CD40 following IFNγ stimulation supports the notion of the brain endothelium being able to present antigens to and co-stimulate T cells promoting effector CD4^+^ T cell responses. Additionally, with constitutively high expression of MHC I, HBEC, like most cell types, possess the minimal requirement for antigen presentation to CD8^+^ T cells.

Antigen uptake is the first step in antigen-presenting pathways, and pinocytosis is the major means by which cells sample soluble protein antigen. Here we show that HBEC are able to take up soluble antigen using both macropinocytosis and clathrin-coated pits as pathways for antigen uptake. Whilst liver sinusoidal EC have been demonstrated to be fully efficient APC in that they express co-stimulatory molecules [30], take up antigen via the mannose receptor [31] and are able to cross present exogenous antigen [32], no previous studies have been conducted on the ability of HBEC to take up and process antigens. The data presented here shows for the first time that HBEC are able to take up soluble antigen using actin-dependent mechanisms, in a manner similar to ‘professional’ APCs.

In the co-culture assays presented here, HBEC were able to support and promote the proliferation of TCR-stimulated CD4^+^ and CD8^+^ T cells. In these assays, an MLR occurs and the T cells proliferate due to an MHC mismatch [33]. The demonstration of antigen-specific activation of human T cells by EC has previously been hampered by the requirement for MHC-matched EC and T cells. Some studies using MHC matched donors support the model that cultured human EC are able to present antigen and activated CD4^+^ T cells [9]–[11]. Moreover, mouse T cell clones or T cells from TCR-transgenic mice can be stimulated to proliferate in a peptide-antigen-specific manner by co-culture with MHC-matched ECs and the relevant protein antigen [14], [34]. Additionally, as presented in this study with our HBEC line, co-cultures of MHC-mismatched EC and T cells result in the activation of CD4^+^ and CD8^+^ T cells demonstrating that EC are able to present alloantigens [15], [16]. In this study we have used a widely accepted assay of allogenic T cell stimulation without well characterised antigens in order to prepare for future experiments that will involve defined malarial antigens. In this assay, the separation of HBEC and T cells resulted in reduced T cell proliferation, indicating the role of cell-cell contact in this phenomenon. The co-stimulatory molecules CD40 and ICOSL are likely to be mediating this effect. ICOSL, a B-7 co-stimulatory family member was upregulated on HBECs following cytokine stimulation. Moreover, ICOSL has been shown previously to be a major co-stimulator in Human umbilical vein EC-mediated T cell activation, particularly in the re-activation of effector/memory T cells [12], [26]. Another co-stimulatory molecule, CD40, was constitutively expressed on HBEC and upregulated after IFNγ stimulation (Fig. 1). CD40 regulates the adhesion of CD4^+^ T cells to brain endothelium via the interaction with its ligand, CD40L on T cells, suggesting a potential mechanism by which activated CD40L expressing T cells could enhance adhesion and migration of inflammatory cells across the BBB to sites of inflammation in the human central nervous system [23].

This increase in HBEC MHC II expression has relevance for CM pathogenesis as MHC II expression on isolated mouse brain EC has been associated with the genetic susceptibility to CM [35]. Moreover, more recently the HLA ligand, HLA-C1 along with its cognate natural killer (NK) cell immunoglobulin-like receptor were shown to be significantly associated with the development of CM in humans [36]. EC, at least from lymph nodes, can be modulators of immune responses as they express multiple peripheral tissue antigens, independent of the autoimmune regulator, AIRE [37], and can even induce anergy [38]. This, together with our observation of malarial antigen transfer to brain EC surfaces [3], opens more possibilities for endothelial-mediated immunopathological mechanisms in CM. The findings described here are not only a major interest for understanding CM pathogenesis but also other neuroinfections involving disruption of endothelial cell barriers such as neurocysticercosis and toxoplasmosis [39], [40].

In summary, we have shown that human brain endothelium cells express molecules important for T cell stimulation and activation including CD40, MHC II and ICOSL. They readily can take up fluorescently labeled antigens via clathrin-coated pits and macropinocytosis. Moreover, these cells are able to bind to and promote the proliferation of allogeneic T cells *in vitro*. Data presented here supports the hypothesis that HBEC are able to act as APC. This is particularly pertinent in neuroinfections such as CM where the diameter of microvessels is smaller than the size of lymphocytes; the lymphocytes are in constant physical contact with the EC surface. Additionally, in the brains of both mice and human with CM, leukocytes (monocytes and T cells) become arrested in brain microvessels [2] providing further means for intimate EC/T cell interactions. It has long been established that CM is a T cell-dependent disease [41], [42], with both CD4^+^ and CD8^+^ T cells playing key roles in CM pathogenesis [43], [44]. Moreover, this cell-cell contact plays an important role in brain endothelial activation [45], as assessed notably by a dramatic increase in plasma levels endothelial microparticles at the time of CM [46]. The data presented here, in combination with our recent demonstration that HBEC can transfer antigens from malarial-infected red blood cells onto their surface, thereby becoming a target for the immune response, provide key evidence for HBEC to act as antigen presenting cells with the presentation of malaria antigens by brain EC to T cells and the potential activation of cytotoxic mechanisms providing a new explanation for CM pathogenesis.

## Supporting Information

Figure S1 Separation of HBEC and PBMC results in a reduction in both CD4^+^ and CD8^+^ T cell proliferation. Graphical representation of fold increase in proliferation of αCD3/CD28 stimulated CD4^+^ and CD8^+^ T cells co-cultured with TNF/IFNγ stimulated HBEC over unstimulated (control) CD4^+^ and CD8^+^ T cell proliferation. Proliferation assessed by CFSE following 6 days of co-culture either in 24 well plates (black bars) or in 0.4 µm transwells (white bars).
(TIF)
