# Correction: The Brain Microvascular Endothelium Supports T Cell Proliferation and Has Potential for Alloantigen Presentation

**DOI:** 10.1371/annotation/971919c7-d831-4c8c-9dbf-3ad7b2c1f667

**Published:** 2013-04-22

**Authors:** Julie Wheway, Stephanie Obeid, Pierre-Olivier Couraud, Valery Combes, Georges E. R. Grau

Our article did not acknowledge that conflicting data have been published examining the ability of rat brain endothelial cells (EC) in presenting antigen to antigen-specific T cells, although the general consensus is that rat brain EC are able to present antigen but are poor inducers of T cell proliferation. This weak antigen presenting capacity may be due to species differences between the rodent and the human model used in this article. We would like to acknowledge this discrepancy as well as the previous publications below:

Pryce G, Male D, Sedgwick J (1989) Antigen presentation in brain: brain endothelial cells are poor stimulators of T-cell proliferation. Immunology 66: 207-212.

Fabry Z, Waldschmidt MM, Moore SA, Hart MN (1990) Antigen presentation by brain microvessel smooth muscle and endothelium. J Neuroimmunol 28: 63-71.

Risau W, Engelhardt B, Wekerle H (1990) Immune function of the blood-brain barrier: incomplete presentation of protein (auto-)antigens by rat brain microvascular endothelium in vitro. J Cell Biol 110: 1757-1766. 

