# Supplementary figures and images for: The Brain Microvascular Endothelium Supports T Cell Proliferation and Has Potential for Alloantigen Presentation
Source: PLoS One. 2013 Jan 8;8(1):e52586. doi: 10.1371/journal.pone.0052586 (PMC3540051; doi:10.1371/journal.pone.0052586)

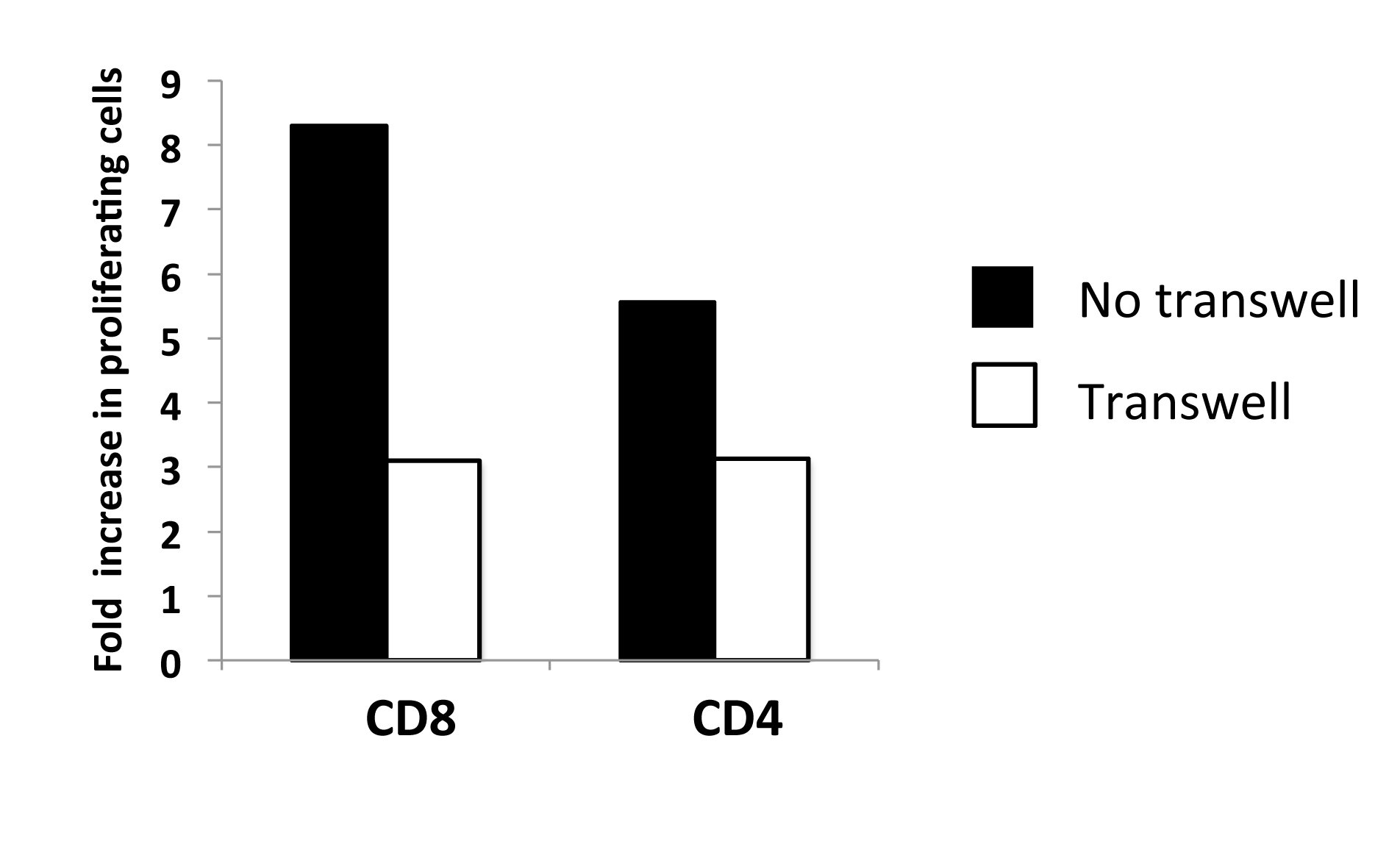

Supplement: Figure S1 — Separation of HBEC and PBMC results in a reduction in both CD4+ and CD8+ T cell proliferation. Graphical representation of fold increase in proliferation of αCD3/CD28 stimulated CD4+ and CD8+ T cells co-cultured with TNF/IFNγ stimulated HBEC over unstimulated (control) CD4+ and CD8+ T cell proliferation. Proliferation assessed by CFSE following 6 days of co-culture either in 24 well plates (black bars) or in 0.4 µm transwells (white bars). (TIF) [file pone.0052586.s001.tif]
